# Epidemiology and Comorbidities of Psychodermatologic Conditions

**DOI:** 10.1177/12034754251347569

**Published:** 2025-06-24

**Authors:** Parsa Abdi, Tarek Turk, Zaim Haq, Michael J. Diaz, Marlene Dytoc

**Affiliations:** 1 Faculty of Medicine, Memorial University, St. Johns, NL, Canada; 2 Faculty of Medicine and Dentistry, University of Alberta, Edmonton, AB, Canada; 3 Warren Alpert Medical School, Brown University, Providence, RI, USA; 4 College of Medicine, University of Florida, Gainesville, FL, USA; 5 Division of Dermatology, Department of Medicine, University of Alberta, Edmonton, AB, Canada

**Keywords:** psychodermatology, trichotillomania, body dysmorphic disorder, skin picking disorder, delusions of parasitosis, dermatitis artefacta, depression, anxiety, phobia

## Abstract

**Introduction:**

Psychodermatologic conditions include primary psychodermatologic disorders (PPDs), psychological conditions manifesting with dermatologic symptoms, and psychophysiological disorders, dermatologic conditions influenced by psychological stress. Despite their clinical significance and considerable impact on quality of life, the comprehensive epidemiology and neuropsychiatric comorbidity profiles of these disorders remain limited.

**Objectives:**

To evaluate the prevalence and comorbidity profiles of psychodermatologic conditions in a diverse, population-based cohort.

**Methods:**

A nested, case–control study was conducted using data from the All of us research program. From 287,011 eligible participants, 984 patients with PPDs (trichotillomania, skin picking disorder, dermatitis artefacta, body dysmorphic disorder, delusions of parasitosis) and 40,535 patients with psychophysiological disorders (psoriasis, atopic dermatitis, acne vulgaris, hidradenitis suppurativa, vulvodynia) were identified using International Classification of Diseases, 10th Revision, Clinical Modification codes. Each patient was paired with 4 controls based on age, sex, and race/ethnicity using nearest-neighbor propensity score matching. Multivariable logistic regression calculated adjusted odds ratios (aOR) and 95% confidence intervals to evaluate associations with various neuropsychiatric comorbidities.

**Results:**

PPDs showed low point prevalences (range: ≤0.01-0.17%) but demonstrated markedly higher odds of neuropsychiatric comorbidities, including depressive disorders (range: aOR, 5.72-13.94), anxiety disorders (range: aOR, 5.96-8.40), and personality disorders (range: aOR, 8.67-13.56 Psychophysiological disorders had higher prevalence rates (range: 0.14%-5.72%) but showed more moderate associations, including depressive disorders (range: aOR, 2.24-3.13), neurodevelopmental disorders (range: aOR, 1.20-2.36), and sleep-wake disorders (range: aOR, 2.25-3.95).

**Conclusions:**

Findings reveal distinct but overlapping comorbidity profiles between PPDs and psychophysiological disorders, emphasizing the need for tailored interventions that address the psychosocial and biological complexities of these conditions.

## Introduction

Psychodermatological conditions are broadly categorized into 3 main groups, namely primary psychiatric disorders with cutaneous symptoms, secondary psychiatric disorders where dermatological diseases result in psychiatric symptoms, and psychophysiological disorders where dermatological conditions are aggravated or precipitated by stress.^1^ Primary psychiatric disorders with cutaneous symptoms also known as primary psychodermatologic disorders (PPDs) encompass conditions where the primary psychopathology results in dermatological manifestations and includes conditions such as delusional parasitosis, trichotillomania, psychogenic pruritus, and pathological skin picking.^2-4^ These disorders are intrinsically linked to psychological disturbances and often present significant diagnostic and therapeutic challenges in both dermatological and psychiatric settings.^4-6^ Psychophysiological disorders, on the other hand, are dermatological conditions that are exacerbated or triggered by psychological stress, such as psoriasis, atopic dermatitis, and acne vulgaris.^1^ The bidirectional relationship between skin conditions and mental health is complex, often creating a vicious cycle where psychological factors aggravate skin conditions, which in turn induce further psychological sequelae.^7,8^ This interplay underscores the necessity for integrated dermatological and psychiatric care.

Despite the significant burden of psychiatric comorbidities among patients with both PPDs and psychophysiological disorders, detailed investigations into the range and frequency of these comorbidities are limited.^9,10^ Existing studies primarily focus on individual conditions or explore psychiatric aspects of specific dermatological disorders, leaving a gap in understanding the comprehensive neuropsychiatric profiles of these patients. The objective of this study is to delineate the epidemiology and neuropsychiatric comorbidities associated with both PPDs and psychophysiological disorders, and to quantify the prevalence of these comorbidities in a large, diverse population. By understanding the neuropsychiatric profiles of patients with these conditions, we aim to highlight the necessity for comprehensive, integrated care approaches that address both dermatological and psychological needs, ultimately improving patient outcomes.

## Methods

### Study Design and Data Source

This study is a nested, case–control analysis following the Strengthening the Reporting of Observational Studies in Epidemiology (STROBE) reporting guidelines, conducted using data from the All of Us (AoU) Research Program. The AoU program is an ongoing National Institutes of Health initiative aimed at creating a comprehensive health database by enrolling populations historically underrepresented in biomedical research.^11^ As of November 2024, the AoU dataset includes data from 844,490 participants, featuring a diverse cohort with a balanced gender distribution, a wide age range, and significant representation from various ethnic and racial groups (18.65% Black, 15.65% Hispanic, and 3.35% Asian). The program collects a wide range of data through self-reported surveys, electronic health records (EHRs), physical measurements, biospecimens, and physical wearables such as Fitbit devices. The Observational Health and Medicines Outcomes Partnership (OMOP) common data model v5.2 developed and upheld by the open-science, Observational Health and Data Sciences Initiative, is utilized to map all data types in this study.

The study protocol was reviewed and approved by the Institutional Review Board of the AoU Research Program. All data used in this study were de-identified to protect participant privacy. Participants provided informed consent for the use of their data in research, and all research activities were conducted in accordance with the principles outlined in the Declaration of Helsinki.^12^

### Participant Population

**Figure 1.**
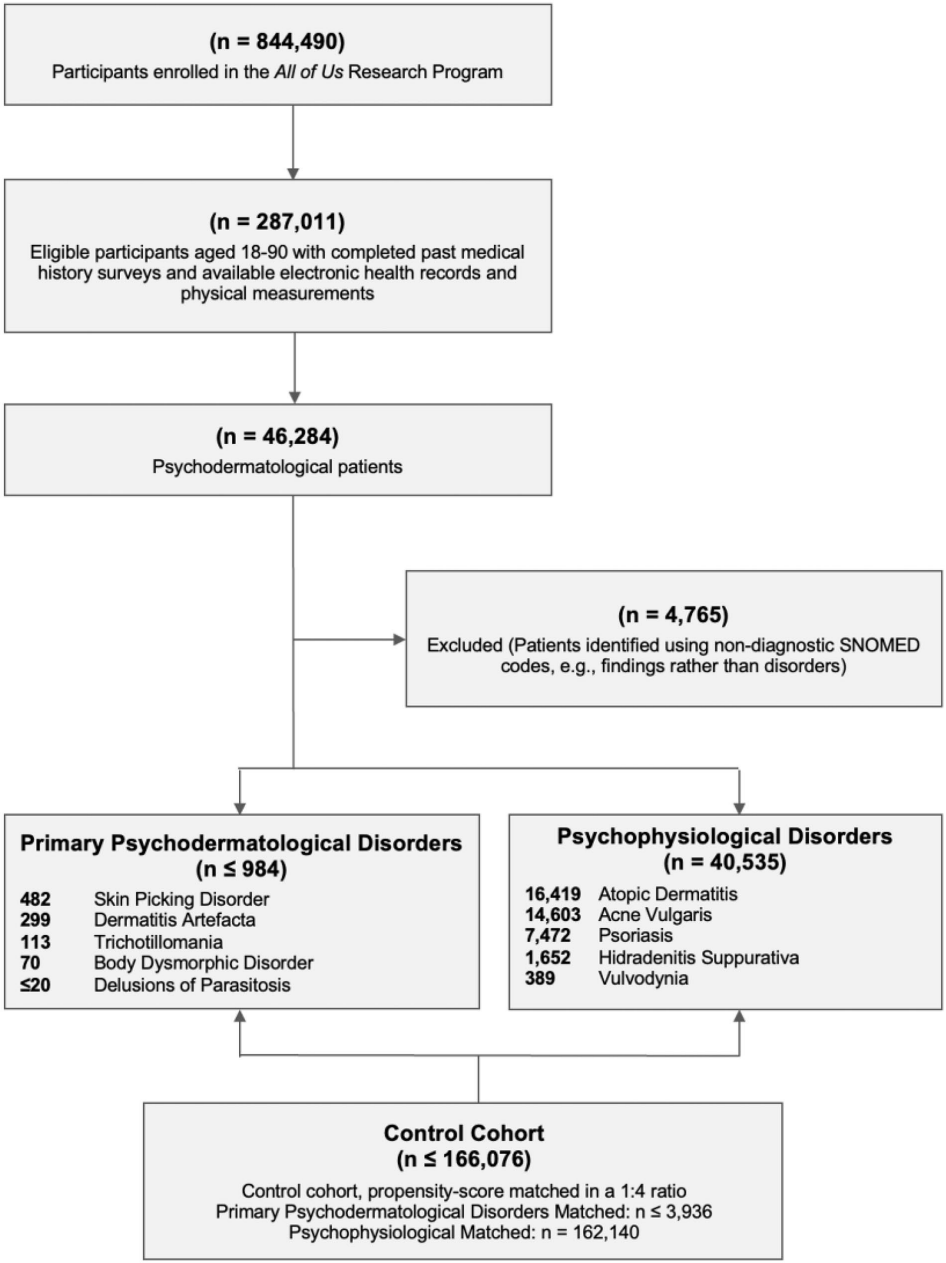
Flow diagram illustrating selection and matching of study cohort. Per All of Us data use standards, values less than 20 are reported as “≤20” to protect participant privacy. Consequently, the total number of primary psychodermatological-related patients is “≤984,” and their control cohort is “≤3936,” based on a 1:4 matching ratio.

Participants in this study were selected from the AoU Research Program database. Inclusion criteria for cases included individuals diagnosed with one or more PPDs, aged 18-90 years of age at the time of initial enrollment, who had completed the relevant sections of the personal medical history survey, had available EHRs, and were diagnosed with one or more of the psychodermatologic conditions of interest. The available PPDs in the AoU database included trichotillomania, skin picking disorder, dermatitis artefacta, body dysmorphic disorder, and delusions of parasitosis. In accordance with the AoU data access policy, PPDs with 20 or fewer participants were included but reported without specific detail to protect individual-level privacy. Psychophysiological disorders included psoriasis, atopic dermatitis, acne vulgaris, hidradenitis suppurativa, and vulvodynia. These conditions were selected based on their high prevalence, well-documented links to psychological stress, and significant burden on quality of life.^13^ Including all psychophysiological disorders was not feasible within the scope of this study due to the breadth of conditions and the substantial variability in their clinical and psychosocial impact. The selected disorders were chosen to represent a diverse range of psychophysiological interactions while maintaining analytical feasibility and focus. Patients identified based on nondiagnostic criteria (e.g., findings rather than confirmed disorders) were excluded (Figure 1).

The cohorts were identified using International Classification of Diseases, 10th Revision, Clinical Modification (ICD-10-CM) codes and Systematized Nomenclature of Medicine (SNOMED) codes specific to each condition (Table S1). For each case, up to 4 control participants were matched using an optimal pair-matching algorithm based on age, sex, and race/ethnicity using nearest-neighbor propensity score matching.^14^ This ratio was selected to enhance the precision of effect sizes without overfitting.^15^

### Variables and Data Extraction

The primary variables of interest were the diagnoses of psychodermatologic conditions and associated neuropsychiatric comorbidities. Neuropsychiatric comorbidities included mood, anxiety, sleep, personality, neurodevelopmental, neurological, and substance use disorders as identified by relevant diagnostic codes (Table S1). Additional variables included demographic information (age, sex, race/ethnicity), socioeconomic status, education level, and body mass index (BMI).

Data were extracted from the EHRs and self-reported surveys available within the AoU database. Diagnostic codes were mapped using the OMOP common data model to ensure consistency across different data sources. To ensure that comorbid conditions were distinct from the psychodermatologic diagnoses, we required separate diagnostic codes for each disorder. This approach prevented the artificial inflation of comorbidity estimates due to overlapping classifications and ensured that each identified comorbid condition reflected a discrete, diagnostically independent entity.

### Statistical Analysis

Comparative statistical analyses were performed utilizing the Pearson χ^2^ test for assessing the association between categorical variables, and Fisher’s exact test was used when expected frequencies were low. Continuous variables were compared using the unpaired *t*-test. Initially, univariate logistic regression was employed to calculate the crude odds ratio (OR) between various neuropsychiatric comorbidities and PPDs to explore possible associations. Following this preliminary analysis, multivariable logistic regression was conducted to adjust for potential confounding factors including age, sex, race/ethnicity, socioeconomic status, education, and BMI, thereby deriving the adjusted odds ratio (aOR). The significance threshold was established at *P* < .05, with 95% confidence intervals (CI) calculated via the Wald method. In cases where the sample size of a psychodermatologic cohort was small, certain neuropsychiatric comorbidities did not meet the criteria for analysis due to the absence of observations in either the psychodermatologic cohort or the control group (ie, nonzero values were required in both groups). This limitation affected the calculation of odds ratios for some comorbidities, particularly in smaller cohorts. As a result, only conditions with sufficient data were included in the multivariable regression analysis for these cohorts.

All data analyses were performed using R software (v 4.3.1), an open-source statistical computing environment developed by the R Core Team, in an integrated Jupyter Notebook environment from the AoU Researcher Workbench, a cloud-based platform that facilitates secure data access and analysis.^16^ Our cohort and dataset construction, along with the code/R commands used for analysis, are documented in the AoU Research Workbench and are available to authorized researchers at AoU or from the corresponding author upon reasonable request.

## Results

### Demographic and Clinical Characteristics

From the 844,490 participants enrolled in the AoU Research Program, 287,011 eligible participants aged 18-90 with completed past medical history surveys, available EHRs, and physical measurements were identified. After applying the inclusion and exclusion criteria, 2 main cohorts were established, namely the PPDs consisting of ≤984 patients, and psychophysiological disorders comprising 40,535 patients. The control cohort included ≤166,076 participants, matched at a 1:4 ratio for PPDs (≤3936 controls) and psychophysiological disorders (162,140 controls; Figure 1).

The PPD cohort included skin picking disorder (n = 482), dermatitis artefacta (n = 299), trichotillomania (n = 113), body dysmorphic disorder (n = 70), and delusions of parasitosis (≤20), with corresponding point prevalences of 0.17%, 0.10%, 0.04%, 0.02%, ≤0.01%, respectively. Across the different PPD cohorts, most patients were female (ranging from 67.13% in skin picking disorder to 78.76% in trichotillomania) and predominantly White (ranging from 55.71% in body dysmorphic disorder to 71.97% in skin picking disorder). The mean ages varied slightly, with dermatitis artefacta patients being the oldest (59.84 ± 14.63 years) and body dysmorphic disorder patients being the youngest (44.24 ± 14.46 years; Table S2). Significant socioeconomic disparities were observed in skin picking disorder and dermatitis artefacta, with lower income and educational levels among PPD patients compared to controls (*P* < .001).

The psychophysiological disorder cohort included patients diagnosed with atopic dermatitis (n = 16,419), acne vulgaris (n = 14,603), psoriasis (n = 7472), hidradenitis suppurativa (n = 1652), and vulvodynia (n = 389), with point prevalences of 5.72%, 5.09%, 2.60%, 0.58%, and 0.14%, respectively. Similar to the PPDs, a majority of patients with psychophysiological disorders were female, particularly in hidradenitis suppurativa (79.78% female) and acne (77.82% female). The racial composition showed a predominance of White patients (60.53% for atopic dermatitis, 57.84% for acne vulgaris, 71.35% for psoriasis, 38.44% for hidradenitis suppurativa, and 63.24% for vulvodynia), with notable proportions of Black patients, especially in hidradenitis suppurativa (36.86%), comparable to their white counterparts. The average ages varied among the conditions, with psoriasis patients being the oldest (62.46 ± 15.12 years) and acne vulgaris patients being the youngest (48.56 ± 16.83 years; Table S3).

### Comorbidities in PPDs

Psychiatric comorbidities were highly prevalent across all cohorts of PPDs, with significant variations in the strength of associations observed. Notably, obsessive-compulsive disorder (OCD), personality disorders, depressive disorders, and anxiety disorders were consistently associated with PPDs, underscoring the psychological comorbidity of these conditions.

Trichotillomania demonstrated strong associations with OCDs (aOR: 55.96, 95% CI: 17.24, 270.00), depressive disorders (aOR: 13.94, 95% CI: 8.37, 24.08), and personality disorders (aOR: 13.56, 95% CI: 5.91, 33.53). Neurodevelopmental disorders were also significantly associated (aOR: 8.24, 95% CI: 4.34, 16.04), while associations with somatoform disorders (aOR: 3.63, 95% CI: 1.30, 9.84), and neurocognitive disorders (aOR: 3.41, 95% CI: 1.47, 7.89) were moderate (Figure S1).

Patients with skin picking disorder also exhibited strong associations with OCD (aOR: 25.09, 95% CI: 11.39, 63.58), depressive disorders (aOR: 6.30, 95% CI: 5.04, 7.91), and personality disorders (aOR: 8.67, 95% CI: 5.21, 14.80). Associations with sleep-wake disorders (aOR: 4.29, 95% CI: 3.44, 5.37) and again neurocognitive disorders (aOR: 2.99, 95% CI: 1.41, 5.73) were significant but more modest (Figure S2).

In patients with dermatitis artefacta, OCDs (aOR: 25.27, 95% CI: 10.26, 76.51) similarly were most prevalent followed by personality disorders (aOR: 11.34, 95% CI: 6.67, 19.97). Interestingly other comorbid somatoform disorders were only the third most prevalent comorbidities (aOR: 6.73, 95% CI: 3.65, 12.71). Eating disorders were not significantly associated in this cohort (aOR: 1.47, 95% CI: 0.85, 3.75, *P* = .2734; Figure S3).

Body dysmorphic disorder demonstrated an exceptionally strong association with eating disorders (aOR: 33.97, 95% CI: 12.62, 90.89) and OCDs (aOR: 48.32, 95% CI: 10.04, 403.55). However, associations with neurocognitive disorders were nonsignificant (aOR: 1.88, 95% CI: 0.79, 4.38, *P* = .1508), and somatoform disorders were not reported (Figure S4).

In the delusions of parasitosis cohort, due to the small sample size, several psychiatric comorbidities could not be analyzed because there were no observations in either the delusions of parasitosis cohort or the control group (ie, nonzero values were required in both groups for calculation). This limitation affected the calculation of odds ratios for many comorbidities, resulting in only 5 associations that met the criteria for analysis. Significant associations were observed only for anxiety disorders (aOR: 8.50, 95% CI: 1.02, 91.91) though with wide confidence intervals, reflecting the reduced statistical power. The comprehensive analysis of neuropsychiatric comorbidities and their aORs for each PPD cohort are detailed in Table S4.

### Comorbidities in Psychophysiological Disorders

Psychophysiological disorders, including atopic dermatitis, hidradenitis suppurativa, acne vulgaris, psoriasis, and vulvodynia, also demonstrated significant neuropsychiatric comorbidities. Sleep-wake, depressive, and personality disorders emerged as consistent associations across these conditions.

Atopic dermatitis was strongly associated with somatoform disorders (aOR: 3.62, 95% CI: 3.26, 4.02), trauma- and stressor-related disorders (aOR: 2.88, 95% CI: 2.76, 3.00), and personality disorders (aOR: 2.85, 95% CI: 2.60, 3.12; Figure S5).

In hidradenitis suppurativa, sleep-wake disorders (aOR: 3.95, 95% CI: 3.52, 4.44), personality disorders (aOR: 3.91, 95% CI: 3.04, 5.03), and depressive disorders (aOR: 3.13, 95% CI: 2.79, 3.50) demonstrated the strongest association. Again, associations with neurocognitive disorders were nonsignificant (aOR: 1.04, 95% CI: 0.72, 1.49, *P* = .8529; Figure S6).

Similarly, psoriasis patients exhibited the strongest association with sleep-wake disorders (aOR: 3.95, 95% CI: 3.52, 4.44). Closely followed depressive (aOR: 2.24, 95% CI: 1.99, 2.42), and trauma- and stressor-related disorders (aOR: 2.11, 95% CI: 1.99, 2.25; Figure S7).

Again, acne patients showed the strongest associations with sleep-wake disorders (aOR: 3.95, 95% CI: 3.52, 4.44). There were also moderate but significant associations with anxiety disorders (aOR: 2.46, 95% CI: 2.35, 2.58), depressive disorders (aOR: 2.30, 95% CI: 2.21, 2.39), and personality disorders (aOR: 2.78, 95% CI: 2.53, 3.06; Figure S8).

Interestingly in vulvodynia, somatoform disorders had the highest relative risk (aOR: 6.13, 95% CI: 3.55, 10.74), followed distantly by trauma- and stressor-related disorders (aOR: 3.99, 95% CI: 3.08, 5.18). Anxiety disorders (aOR: 2.77, 95% CI: 2.12, 3.62) and depressive disorders (aOR: 2.39, 95% CI: 1.88, 3.04) also demonstrated significant associations. Several neuropsychiatric comorbidities including, substance use, schizophrenic, neurocognitive, neurodevelopmental, bipolar, and eating disorders were nonsignificant (Figure S9).

The comprehensive analysis of neuropsychiatric comorbidities and their aORs for each PPD cohort are detailed in Table S5.

## Discussion

This study provides a comprehensive, population-based examination of the epidemiology, demographic characteristics, and neuropsychiatric of psychodermatologic conditions, highlighting shared and distinct clinical profiles. Our findings reaffirm the substantial burden of neuropsychiatric comorbidities associated with PPDs as well as several psychophysiological disorders.^17^

We found that PPDs, namely, skin picking disorder, dermatitis artefacta, trichotillomania, body dysmorphic disorder, and delusions of parasitosis had point prevalences of 0.17%, 0.10%, 0.04%, 0.02%, ≤0.01%, respectively. These rates were notably lower than the prevalence rates observed in previous community-recruited studies with smaller sample sizes.^18^ For instance, skin picking disorder has been reported to affect between 1.2% and 11.2% of the general population, while trichotillomania prevalence in community-based studies ranges from 0.6% to 2.9%.^18-21^ Similarly, body dysmorphic disorder has been estimated to range from 0.5% to 3.2% in community settings, significantly higher than our findings.^22-24^ These discrepancies likely arise from methodological rigor, broader population sampling, and stringent inclusion criteria. Unlike many earlier investigations, our approach employed a large, heterogeneous cohort and a structured informatics framework designed to mitigate sampling biases and overestimations of subclinical cases including sampling from only cosmetic or psychiatric cohorts.^25^ When examining prevalence estimates for delusions of parasitosis, which have generally been derived from large, population-based studies, our point prevalence aligns more closely with the reported ranges of 0.004%-0.03%.^26,27^

Our study also found a significant female predominance across several PPDs, which aligns with much of the existing literature on conditions such as skin-picking disorder, trichotillomania, and body dysmorphic disorder.^28-30^ However, variations exist in research, with some studies suggesting more gender parity, especially for trichotillomania.^31^ This discrepancy may be due to underreporting among men, who may be less likely to seek treatment or express distress, often attributing symptoms like hair loss to natural causes rather than behavioral issues. Additionally, sociocultural norms may play a role, with women more likely to report appearance-related concerns, which could contribute to higher diagnosed rates among females.^32^

Importantly, the comorbidity profiles of PPDs reveal a notable convergence of neuropsychiatric conditions. Our findings are generally in keeping with the previously literature suggesting that PPDs frequently co-occur with a wide range of psychiatric conditions, particularly, depression, anxiety, and personality disorders.^9,31,33-37^ Conditions such as dermatitis artefacta and body dysmorphic disorder were strongly associated with OCDs and personality disorders, while also manifesting substantial co-occurrence with depressive and anxiety disorders. Trichotillomania and skin picking disorders, 2 prototypical body-focused repetitive behavior (BFRB) disorders showed notably similar comorbidity profiles and demonstrated robust associations with obsessive-compulsive spectrum conditions, sleep, depressive, and trauma/stress disorders. Collectively, these patterns suggest that PPDs may share underlying dimensions of impulsivity, compulsivity, and affective dysregulation, possibly driven by aberrant cortico–striato–thalamo–cortical circuitry and altered stress–response mechanisms.^38-40^

Psychophysiological disorders also displayed consistent associations with an array of neuropsychiatric conditions but typically to a lower extent. The smaller relative risk observed in our study likely reflects both methodological and intrinsic differences between these conditions. The larger sample sizes of psychophysiological cohorts yielded narrower confidence intervals and more precise estimates, reducing the risk of overestimation seen in smaller PPD cohorts. Moreover, PPDs are fundamentally rooted in psychopathology, where dermatologic symptoms are secondary manifestations of underlying neuropsychiatric processes, resulting in higher rates of psychiatric comorbidities. In contrast, psychophysiological disorders, while significantly associated with psychiatric disorders, typically exhibit these comorbidities as secondary to the psychosocial burden of chronic skin disease rather than as primary drivers.^41-43^ Additionally, differences in diagnostic pathways may also play a role as patients with PPDs are more likely to be evaluated in psychiatric or multidisciplinary settings, where comprehensive psychiatric assessment identifies a broader range of comorbidities.^44^

Interestingly, many of the psychophysiological disorders demonstrated distinctive comorbidity clusters with strong associations for with sleep-wake, personality, and mood disorders—patterns suggestive of a complex stress–responsive diathesis, wherein cutaneous inflammation, chronic pain, and sleep disturbances may synergistically exacerbate psychological distress. Of particular note, vulvodynia displayed a distinct cluster of comorbidities, with exceedingly high odds for somatoform disorders, followed by trauma- and stressor-related conditions. Such a pattern may reflect the complexity of chronic pain syndromes, where psychosocial stress, past traumatic experiences, and heightened somatic sensitivity interact to perpetuate a cycle of distress and dermatologic discomfort.^45,46^

These findings also highlight the significance of shared pathophysiological mechanisms, such as chronic inflammation, hypothalamic–pituitary–adrenal axis dysregulation, and altered neurotransmitter signaling, in linking cutaneous symptoms with neuropsychiatric sequelae.^47,48^ Future studies should focus on clarifying these interactions to guide the development of precise, targeted therapies. For example, medications such as memantine, traditionally employed in neurocognitive disorders, have shown preliminary benefit in certain obsessive-compulsive spectra and BFRBs such as trichotillomania.^49^ Whether such agents might alleviate PPD symptomatology or mitigate stress exacerbation in psychophysiological disorders deserves systematic investigation.

Several limitations must be noted. First, the reliance on ICD-10-CM and SNOMED diagnostic codes and self-reported histories introduces potential misclassification bias, as clinical interviews are the gold standard for diagnosis. Second, despite the large underlying cohort, the relatively modest sample sizes for specific PPDs, especially delusions of parasitosis, limited statistical power and precluded more granular analyses. Third, this cross-sectional design cannot establish causality or directionality of associations. Finally, the inherent heterogeneity in dermatological conditions and their treatment histories may introduce residual confounding.

## Conclusion

Prospective, longitudinal studies are warranted to elucidate the temporal relationships and shared neurobiological mechanisms underpinning these comorbidities. Randomized controlled trials investigating integrated dermatological and psychiatric interventions—potentially including psychopharmacotherapy, cognitive-behavioral therapy, and stress-reduction strategies—could offer clearer guidance for clinicians. Moreover, exploring the impact of novel treatments, including neuromodulatory agents, could uncover therapeutic targets that alleviate both dermatologic and neuropsychiatric burdens. Such endeavors would not only refine our understanding of psychodermatology but also pave the way for precision medicine approaches that attend to the individual’s psychological, biological, and socioenvironmental contexts.

## Supplemental Material

sj-docx-1-cms-10.1177_12034754251347569 – Supplemental material for Epidemiology and Comorbidities of Psychodermatologic Conditions Supplemental material, sj-docx-1-cms-10.1177_12034754251347569 for Epidemiology and Comorbidities of Psychodermatologic Conditions by Parsa Abdi, Tarek Turk, Zaim Haq, Michael J. Diaz and Marlene Dytoc in Journal of Cutaneous Medicine and Surgery

sj-docx-2-cms-10.1177_12034754251347569 – Supplemental material for Epidemiology and Comorbidities of Psychodermatologic Conditions Supplemental material, sj-docx-2-cms-10.1177_12034754251347569 for Epidemiology and Comorbidities of Psychodermatologic Conditions by Parsa Abdi, Tarek Turk, Zaim Haq, Michael J. Diaz and Marlene Dytoc in Journal of Cutaneous Medicine and Surgery
