# Supplementary material for: Epidemiology and Comorbidities of Psychodermatologic Conditions
Source: J Cutan Med Surg. 2025 Jun 24;30(1):33–40. doi: 10.1177/12034754251347569 (PMC12906613; doi:10.1177/12034754251347569)
Supplement: sj-docx-1-cms-10.1177_12034754251347569 – Supplemental material for Epidemiology and Comorbidities of Psychodermatologic Conditions [file sj-docx-1-cms-10.1177_12034754251347569.docx]

**Table S1**: Diagnostic codes from the All of Us research program database used to identify psychodermatologic conditions and associated neuropsychiatric comorbidities.
OMOP CDM; Observational Medical Outcomes Partnership Common Data Model, SNOMED; Systemized Nomenclature of Medicine

| **Category** | **Sub-category** | **SNOMED Code** | **OMOP CDM Concept ID** |
| --- | --- | --- | --- |
| **Primary Psychodermatologic Disorders** | Trichotillomania | 17155009 | 4062972 |
|  | Skin Picking Disorder | 238958004 | 4033045 |
|  | Dermatitis Artefacta | 27720003 | 133285 |
|  | Delusions of Parasitosis | 238973003 | 4081257 |
|  | Body Dysmorphic Disorder | 83482000 | 4306640 |
| **Psychophysiological Disorders** | Psoriasis | 9014002 | 140168 |
|  | Atopic Dermatitis | 24079001 | 133834 |
|  | Hidradenitis Suppurativa | 59393003 | 4241223 |
|  | Acne Vulgaris | 11381005 | 141095 |
|  | Vulvodynia | 238968009 | 436741 |
| **Obsessive-Compulsive Disorders** | Obsessive-Compulsive Disorder | 191736004 | 440374 |
|  | Hoarding Disorder | 247968005 | 4084868 |
| **Personality Disorders** | Cluster A Personality Disorder | 16805009 | 4043918 |
|  | Cluster B Personality Disorder | 4306003 | 4181019 |
|  | Cluster C Personality Disorder | 83890006 | 4222618 |
| **Anxiety Disorders** | Generalized Anxiety Disorder | 21897009 | 434613 |
|  | Panic Disorder | 371631005 | 436074 |
|  | Phobic Disorder | 386810004 | 4304010 |
| **Depressive Disorders** | Major Depressive Disorder | 370143000 | 4152280 |
|  | Persistent Depressive Disorder | 78667006 | 433440 |
|  | Premenstrual Dysphoric Disorder | 596004 | 4242733 |
|  | Disruptive Mood Dysregulation Disorder | 715924009 | 37396201 |
| **Schizophrenia Spectrum** | Schizophrenia | 58214004 | 435783 |
|  | Schizophreniform Disorder | 88975006 | 444434 |
|  | Brief Psychotic Disorder | 5464005 | 4182683 |
|  | Schizoaffective Disorder | 68890003 | 4286201 |
| **Neurodevelopmental Disorders** | Attention-Deficit/Hyperactivity Disorder | 406506008 | 438409 |
|  | Autism Spectrum Disorder | 35919005 | 439776 |
|  | Intellectual Disability | 110359009 | 40277917 |
| **Somatoform Disorders** | Somatization Disorder | 397923000 | 441545 |
|  | Undifferentiated Somatoform Disorder | 18393005 | 434625 |
|  | Illness Anxiety Disorder | 162396005 | 4348945 |
|  | Conversion Disorder | 20734000 | 4049367 |
| **Trauma- and Stressor-Related Disorders** | Post-Traumatic Stress Disorder | 47505003 | 436676 |
|  | Acute Stress Disorder | 67195008 | 440083 |
|  | Adjustment Disorders | 17226007 | 436677 |
| **Eating Disorders** | Anorexia Nervosa | 56882008 | 436675 |
|  | Bulimia Nervosa | 78004001 | 438407 |
|  | Pica | 14077003 | 437839 |
|  | Rumination Disorder | 37941009 | 4242221 |
|  | Other Specified Feeding/Eating Disorder | 72366004 | 439002 |
| **Neurocognitive Disorders** | Delirium | 2776000 | 373995 |
|  | Major Neurocognitive Disorder | 52448006 | 4182210 |
|  | Mild Neurocognitive Disorder | 386805003 | 4297400 |
| **Sleep-Wake Disorders** | Insomnia Disorder | 193462001 | 436962 |
|  | Hypersomnolence Disorder | 77692006 | 438134 |
|  | Narcolepsy | 60380001 | 436100 |
|  | Breathing-Related Sleep Disorders | 39898005 | 435524 |
|  | Parasomnia | 58690002 | 440087 |
| **Bipolar Disorders** | Bipolar I Disorder | 371596008 | 432876 |
|  | Bipolar II Disorder | 83225003 | 4307956 |
|  | Cyclothymic Disorder | 76105009 | 440696 |
| **Substance Use Disorder** | Alcohol Use Disorder | 15167005 | 433753 |
|  | Opioid Use Disorder | 5602001 | 438130 |
|  | Cannabis Use Disorder | 37344009 | 434327 |
|  | Stimulant Use Disorder | 441527004 | 40479573 |
|  | Inhalant Use Disorder | 70340006 | 4290538 |
|  | Sedative/Hypnotic/Anxiolytic Use Disorder | 268640002 | 443236 |

**Table S2**: Sociodemographic and clinical traits of primary psychodermatologic disorder patients in the All of Us Research Program.
Note: Patients with Delusions of Parasitosis (≤20) are excluded from this table to adhere to the data use standards of the All of Us Research Program. Additionally, categories with participant counts ≤20 are suppressed ("≤20") across all characteristics to ensure the protection of participant confidentiality.
SPD: skin picking disorder, DA: dermatitis artefacta, BDD: body dysmorphic disorder, TTM: trichotillomania, Ref: reference, No: number, SD: standard deviation, HS: high school

| **Characteristic** | **SPD Cases**  **No. (%)**  **(n= 482)** | **DA Cases**  **No. (%)**  **(n= 299)** | **BDD Cases**  **No. (%)**  **(n= 70)** | **TTM Cases**  **No. (%)**  **(n=113)** |
| --- | --- | --- | --- | --- |
| Age (SD) ^†^ | 53.85 (15.79) | 59.84 (14.63) | 44.24 (14.46) | 46.01 (16.23) |
| Sex^†^ |  |  |  |  |
| Male | 145 (30.10) | 74 (24.75) | 21 (30.00) | 24 (21.24) |
| Female/Other^1^ | 337 (69.90) | 216 (72.24) | 49 (70.00) | 85 (78.76) |
| Race/Ethnicity^†^ |  |  |  |  |
| White | 347 (71.97) | 215 (71.91) | 39 (55.71) | 64 (56.64) |
| Asian/Hispanic | 37 (7.68) | 28 (9.36) | ≤20 | ≤20 |
| Black | 60 (12.46) | 32 (10.70) | ≤20 | 21 (18.58) |
| Other^2^ | 38 (7.96) | 24 (8.03) | ≤20 | ≤20 |
| Annual Income |  |  |  |  |
| ≥ $50k | 155 (32.18) | 92 (30.77) | 25 (35.71) | 37 (32.74) |
| $10k- $50k | 152 (31.58) | 99 (33.11) | 26 (37.14) | 39 (34.51) |
| ≤ $10k | 112 (23.18) | 53 (17.73) | ≤20 | ≤20 |
| Other^3^ | 63 (13.15) | 55 (18.39) | ≤20 | ≤20 |
| Education |  |  |  |  |
| College graduate | 158 (32.87) | 115 (38.46) | 32 (45.71) | 56 (49.56) |
| Attended college, no degree | 171 (35.47) | 103 (34.45) | 21 (30.00) | 26 (23.01) |
| 12^th^ Grade | 102 (21.11) | 54 (18.06) | ≤20 | ≤20 |
| No HS degree | 30 (6.23) | ≤20 | ≤20 | ≤20 |
| Other^3^ | 21 (4.36) | ≤20 | ≤20 | ≤20 |
|  |  |  |  |  |

^†^Indicates variable propensity matched with control cohort.

^1^Includes nonbinary, transgender, and other gender identity.

^2^Includes Middle Eastern or North African, American Indian or Alaska Native, Hawaiian or another Pacific Islander, and other race and ethnicity.

^3^Includes participants who preferred not to answer the question.

**Table S3:** Sociodemographic and clinical traits of psychophysiological disorder patients in the All of Us Research Program.
**Note:** Categories with participant counts ≤20 are suppressed ("≤20") across all characteristics to ensure the protection of participant confidentiality and to adhere to the data use standards of the All of Us Research Program.
AD: atopic dermatitis, HS: hidradenitis suppurativa, Ref: reference, No: number, SD: standard deviation

| **Characteristic** | **AD Cases**  **No. (%)**  **(n= 16,419)** | **Acne Cases**  **No. (%)**  **(n= 14,603)** | **Psoriasis Cases**  **No. (%)**  **(n= 7,472)** | **HS Cases**  **No. (%) (n=1,652)** | **Vulvodynia Cases**  **No. (%)**  **(n=389)** |
| --- | --- | --- | --- | --- | --- |
| Age (SD) ^†^ | 60.94 (16.27) | 48.56 (16.83) | 62.46 (15.12) | 47.81 (14.16) | 53.28 (16.94) |
| Sex^†^ |  |  |  |  |  |
| Male | 5385 (32.80) | 2992 (20.49) | 2881 (38.56) | 302 (18.28) | 0 (0) |
| Female | 10,682 (65.06) | 11,364 (77.82) | 4412 (59.05) | 1318 (79.78) | 389 (100.00)^1*^ |
| Other^1^ | 352 (2.14) | 247 (1.69) | 179 (2.40) | 32 (1.94) | 0 (0) |
| Race/Ethnicity^†^ |  |  |  |  |  |
| White | 9938 (60.53) | 8446 (57.84) | 5331 (71.35) | 635 (38.44) | 246 (63.24) |
| Asian | 501 (3.05) | 524 (3.59) | 155 (2.07) | 22 (1.33) | ≤20 |
| Black | 2806 (17.09) | 2337 (16.00) | 557 (7.45) | 609 (36.86) | 50 (12.85) |
| Hispanic | 1909 (11.63) | 1947 (13.33) | 818 (10.95) | 226 (13.68) | 58 (14.91) |
| Other^2^ | 1265 (7.70) | 1349 (9.24) | 611 (8.18) | 160 (9.67) | ≤20 |
| Annual Income |  |  |  |  |  |
| ≥ $50k | 7532 (45.87) | 6959 (47.65) | 3477 (46.53) | 381 (23.06) | 187 (48.07) |
| $35k- $50k | 1381 (8.41) | 1376 (9.42) | 657 (8.79) | 153 (9.26) | 33 (8.48) |
| $25k- $35k | 1102 (6.71) | 1027 (7.03) | 534 (7.15) | 139 (8.41) | 21 (5.40) |
| $10k- $25k | 1855 (11.30) | 1408 (9.64) | 871 (11.66) | 283 (17.13) | 52 (13.37) |
| ≤ $10k | 1629 (9.92) | 1386 (9.49) | 613 (8.20) | 375 (22.70) | 34 (8.74) |
| Other^3^ | 2920 (17.78) | 2447 (16.76) | 1320 (17.67) | 321 (19.43) | 62 (15.93) |
| Education |  |  |  |  |  |
| College graduate | 8291 (50.50) | 8071 (55.27) | 3800 (50.86) | 456 (27.60) | 207 (53.21) |
| Attended college, no degree | 4102 (24.98) | 3662 (25.08) | 1962 (26.26) | 595 (36.02) | 99 (25.45) |
| 12^th^ Grade | 2561 (15.60) | 1964 (13.45) | 1107 (14.82) | 404 (24.46) | 45 (11.57) |
| No HS degree | 1001 (6.10) | 558 (3.82) | 410 (5.49) | 150 (9.08) | 38 (9.78)^3*^ |
| Other^3^ | 464 (2.83) | 348 (2.38) | 193 (2.58) | 47 (2.85) | 0 (0) |
|  |  |  |  |  |  |

^†^Indicates variable propensity matched with control cohort.

^1^Includes nonbinary, transgender, and other gender identity.
^1*^For vulvodynia patients, nonbinary, transgender, and other gender identities have been aggregated into the Female category to protect participant confidentiality.

^2^Includes Middle Eastern or North African, American Indian or Alaska Native, Hawaiian or another Pacific Islander, and other race and ethnicity.

^3^Includes participants who preferred not to answer the question.
^3*^For vulvodynia patients, nonbinary, transgender, and other gender identities have been aggregated into the Female category to protect participant confidentiality.

**Table S4:** Multivariable-adjusted odds ratios for neuropsychiatric comorbidities among primary psychodermatology patients.

| **Disease** | **Skin Picking Disorder**  **aOR (95% CI)†** | **Dermatitis Artefacta aOR (95% CI)†** | **Body Dysmorphic Disorder aOR (95% CI)†** | **Trichotillomania  aOR (95% CI)†** | **Delusional parasitosis aOR (95% CI)†** |
| --- | --- | --- | --- | --- | --- |
| Obsessive-Compulsive Disorders | 25.09 (11.39, 63.58)** | 25.27 (10.26, 76.51)** | 48.32 (10.04, 403.55)** | 55.96 (17.24, 270.00)** | NA |
| Personality Disorder | 8.67 (5.21, 14.80)** | 11.34 (6.67, 19.97)** | 12.34 (7.31, 21.62)** | 13.56 (5.91, 33.53)** | 4.75 (0.16, 139.21)^ns^ |
| Anxiety Disorders | 5.96 (4.71, 754)** | 6.08 (4.50, 8.24)** | 8.29 (4.41, 15.94)** | 8.40 (5.18, 13.85)** | 8.50 (1.02, 91.91)* |
| Depressive Disorders | 6.30 (5.04, 7.91) ** | 5.72 (4.33, 7.58)** | 12.62 (6.69, 25.12)** | 13.94 (8.37, 24.08)** | 1.24 (0.14, 9.32)^ns^ |
| Schizophrenia Spectrum | 5.83 (2.64, 13.35)** | 3.93 (1.90, 8.21)* | 3.93 (1.90, 8.21)* | 6.35 (1.26, 36.31)* | 4.65 (0.22, 130.38)^ns^ |
| Neurodevelopmental | 5.47 (3.85, 7.80)** | 5.64 (3.58, 8.93)** | 2.45 (0.81, 6.93)^ns^ | 8.24 (4.34, 16.04)** | NA |
| Somatoform Disorders | 5.70 (3.26, 10.10)** | 6.73 (3.65, 12.71)** | NA | 3.63 (1.30, 9.84)** | NA |
| Trauma- and Stressor-Related Disorders | 4.79 (3.80, 6.03)** | 6.29 (4.70, 8.43)** | 7.12 (4.02, 12.79)** | 5.60 (3.44, 9.19)** | NA |
| Eating Disorders | 5.42 (2.49, 11.97)** | 1.47 (0.85, 3.75)^ns^ | 33.97 (12.62, 90.89)** | 4.51 (1.73, 11.93)** | NA |
| Neurocognitive Disorders | 2.99 (1.41, 5.73)** | 2.54 (1.29, 4.78)* | 1.88 (0.79, 4.38)^ns^ | 3.41 (1.47, 7.89)** | NA |
| Sleep-Wake Disorders | 4.29 (3.44, 5.37)* | 4.23 (3.21, 5.61)** | 4.59 (2.58, 8.28)** | 5.27 (3.30, 8.51)** | 2.25 (0.31, 20.20)^ns^ |
| Bipolar Disorders | 4.16 (2.79, 6.20)** | 5.61 (3.77, 8.40)** | 3.88 (1.80, 8.40)* | 4.46 (2.21, 9.05)** | NA |
| Substance Use Disorders | 4.30 (2.98, 6.22)** | 4.04 (2.79, 5.87)** | 2.53 (1.16, 5.42)* | 4.81 (2.68, 8.70)** | NA |
|  |  |  |  |  |  |

*aOR*: multivariable adjusted odds ratio, *CI*: confidence interval
NA: Not Available due to insufficient sample size or lack of observations.

†aOR: Multivariable regression analysis controlled for age, race, ethnicity, sex, income, body mass index, and education.
** p < 0.001, * p < 0.05, ns non-significant.

**Table S5**: Multivariable-adjusted odds ratios for neuropsychiatric comorbidities among patients with psychophysiological disorders.

| **Disease** | **Psoriasis aOR (95% CI)†** | **Atopic Dermatitis aOR (95% CI)†** | **Hidradenitis Suppurativa aOR (95% CI)†** | **Acne Vulgaris aOR (95% CI)†** | **Vulvodynia aOR (95% CI)†** |
| --- | --- | --- | --- | --- | --- |
| Obsessive-Compulsive Disorders | 2.05 (1.62, 2.58)** | 2.81 (2.41, 3.28)** | 2.67 (1.62, 4.33)* | 3.02 (2.60, 3.50)** | 3.04 (1.28, 6.98)* |
| Personality Disorder | 2.10 (1.82, 2.41)** | 2.85 (2.60, 3.12)** | 3.91 (3.04, 5.03)** | 2.78 (2.53, 3.06)** | 3.51 (2.03, 6.02)** |
| Anxiety Disorders | 2.03 (1.90, 2.18)** | 2.61 (2.49, 2.73)** | 2.64 (2.30, 3.02)** | 2.46 (2.35, 2.58)** | 2.77 (2.12, 3.62)** |
| Depressive Disorders | 2.24 (1.99, 2.42)** | 2.28 (2.19, 2.36)** | 3.13 (2.79, 3.50)** | 2.30 (2.21, 2.39)** | 2.39 (1.88, 3.04)** |
| Schizophrenia Spectrum | 1.82 (1.47, 2.25)** | 2.02 (1.77, 2.30)** | 2.22 (1.58, 3.10)** | 1.26 (0.98, 1.42)^ns^ | 1.11 (0.79, 5.15)^ns^ |
| Neurodevelopmental | 1.52 (1.20, 2.06)* | 2.36 (2.18, 2.56)** | 2.33 (1.88, 2.89)** | 1.60 (1.43, 1.79)** | 1.20 (0.76, 2.57)^ns^ |
| Somatoform Disorders | 1.99 (1.69, 2.34)** | 3.62 (3.26, 4.02)** | 2.61 (1.85, 3.66)** | 3.13 (2.80, 3.50)** | 6.13 (3.55, 10.74)** |
| Trauma- and Stressor-Related Disorders | 2.11 (1.99, 2.25)** | 2.88 (2.76, 3.00)** | 2.75 (2.42, 3.13)** | 3.09 (2.96, 3.23)** | 3.99 (3.08, 5.18)** |
| Eating Disorders | 1.75 (1.31, 2.08)* | 2.21 (1.95, 2.50)** | 2.17 (1.51, 3.10)** | 2.34 (2.08, 2.62)** | 1.78 (0.92, 3.87)^ns^ |
| Neurocognitive Disorders | 1.34 (1.07-1.68)* | 2.12 (1.92-2.35)** | 1.04 (0.72-1.49)^ns^ | 1.10 (0.89-1.45)^ns^ | 1.12 (0.61-2.00)^ns^ |
| Sleep-Wake Disorders | 2.25 (2.14, 2.37)** | 2.63 (2.54, 2.73)** | 3.95 (3.52, 4.44)** | 2.48 (2.38, 2.58)** | 2.68 (2.11, 3.41)** |
| Bipolar Disorders | 1.82 (1.64, 2.01)** | 2.02 (1.89, 2.17)** | 2.71 (2.29, 3.21)** | 1.68 (1.56, 1.81)** | 1.67 (0.92, 2.76)^ns^ |
| Substance Use Disorders | 1.74 (1.60, 1.89)** | 1.76 (1.66, 1.86)** | 2.39 (2.06, 2.77)** | 1.55 (1.45, 1.65)** | 1.10 (0.68, 1.71)^ns^ |
|  |  |  |  |  |  |

*aOR*: multivariable adjusted odds ratio, *CI*: confidence interval
NA: Not Available due to insufficient sample size or lack of observations.

†aOR: Multivariable regression analysis controlled for age, race, ethnicity, sex, income, body mass index, and education.
** p < 0.001, * p < 0.05, ns = non-significant
