# Supplementary material for: Epidemiology and Comorbidities of Psychodermatologic Conditions
Source: J Cutan Med Surg. 2025 Jun 24;30(1):33–40. doi: 10.1177/12034754251347569 (PMC12906613; doi:10.1177/12034754251347569)
Supplement: sj-docx-2-cms-10.1177_12034754251347569 – Supplemental material for Epidemiology and Comorbidities of Psychodermatologic Conditions [file sj-docx-2-cms-10.1177_12034754251347569.docx]

**Figure S1**: Multivariable-adjusted odds ratios (aOR) for psychiatric comorbidities in patients with trichotillomania. The dashed line represents the significance threshold (aOR = 1). Green bars indicate statistically significant associations (p < 0.05), while red bars represent non-significant associations (p > 0.05). Error bars represent 95% confidence intervals.

**Figure S2**: Multivariable-adjusted odds ratios (aOR) for psychiatric comorbidities in patients with skin picking disorder. The dashed line represents the significance threshold (aOR = 1). Green bars indicate statistically significant associations (p < 0.05), while red bars represent non-significant associations (p > 0.05). Error bars represent 95% confidence intervals.

**Figure S3**: Multivariable-adjusted odds ratios (aOR) for psychiatric comorbidities in patients with dermatitis artefacta. The dashed line represents the significance threshold (aOR = 1). Green bars indicate statistically significant associations (p < 0.05), while red bars represent non-significant associations (p > 0.05). Error bars represent 95% confidence intervals.

**Figure S4**: Multivariable-adjusted odds ratios (aOR) for psychiatric comorbidities in patients with body dysmorphic disorder. The dashed line represents the significance threshold (aOR = 1). Green bars indicate statistically significant associations (p < 0.05), while red bars represent non-significant associations (p > 0.05). Error bars represent 95% confidence intervals.

**Figure S5**: Multivariable-adjusted odds ratios (aOR) for psychiatric comorbidities in patients with atopic dermatitis. The dashed line represents the significance threshold (aOR = 1). Green bars indicate statistically significant associations (p < 0.05), while red bars represent non-significant associations (p > 0.05). Error bars represent 95% confidence intervals.

**Figure S6**: Multivariable-adjusted odds ratios (aOR) for psychiatric comorbidities in patients with hidradenitis suppurativa. The dashed line represents the significance threshold (aOR = 1). Green bars indicate statistically significant associations (p < 0.05), while red bars represent non-significant associations (p > 0.05). Error bars represent 95% confidence intervals.

**Figure S7**: Multivariable-adjusted odds ratios (aOR) for psychiatric comorbidities in patients with psoriasis. The dashed line represents the significance threshold (aOR = 1). Green bars indicate statistically significant associations (p < 0.05), while red bars represent non-significant associations (p > 0.05). Error bars represent 95% confidence intervals.

**Figure S8**: Multivariable-adjusted odds ratios (aOR) for psychiatric comorbidities in patients with acne vulgaris. The dashed line represents the significance threshold (aOR = 1). Green bars indicate statistically significant associations (p < 0.05), while red bars represent non-significant associations (p > 0.05). Error bars represent 95% confidence intervals.

**Figure S9**: Multivariable-adjusted odds ratios (aOR) for psychiatric comorbidities in patients with vulvodynia. The dashed line represents the significance threshold (aOR = 1). Green bars indicate statistically significant associations (p < 0.05), while red bars represent non-significant associations (p > 0.05). Error bars represent 95% confidence intervals.
